# The role of individual and contextual economic factors in obesity among adolescents: A cross-sectional study including 143 160 participants from 41 countries

**DOI:** 10.7189/jogh.14.04035

**Published:** 2024-02-23

**Authors:** Alejandra Gallego, José Francisco López-Gil

**Affiliations:** 1 Department of Applied Economics, Faculty of Economics and Business, University of Murcia, Murcia, Spain; 2 One Health Research Group, Universidad de Las Américas, Quito, Ecuador

## Abstract

**Background:**

To our knowledge, no previous study has examined the role of index of economic freedom (IEF) in the prevalence of excess weight and obesity in adolescents. The aim of this study was to determine the association between both individual and contextual economic factors and the prevalence of overweight and obesity (i.e. excess weight) or obesity in adolescents from different countries.

**Methods:**

A cross-sectional study was carried out using data from the 2017/2018 wave of the Health Behaviour School-Aged Children study. Body mass index z-score was determined following the International Obesity Task Force criteria and, subsequently, excess weight and obesity were computed. The Family Affluence Scale was used to assess socioeconomic status. The index of IEF was used to estimate the benefits of economic freedom, both for individuals and for society as a whole.

**Results:**

An inverse association was shown between socioeconomic status (SES) and excess weight or obesity, with adolescents with high SES and medium SES being less likely to have excess weight compared to adolescents with low SES (medium SES: odds ratio (OR) = 0.79; 95% confidence interval (CI) = 0.77–0.82, *P* < 0.001; high SES: OR = 0.65; 95% CI = 0.62–0.68, *P* < 0.001). For obesity, lower odds were also found for adolescents with medium SES (medium SES: OR = 0.74; 95% CI = 0.69–0.80, *P* < 0.001) or high SES (high SES: OR = 0.55; 95% CI 0.49–0.61, *P* < 0.001), in comparison with their counterparts with low SES. On the other hand, it was observed a lower likelihood of having excess weight and obesity in mostly unfree countries (excess weight: OR = 0.72; 95% CI = 0.51–1.00, *P* = 0.052; obesity: OR = 0.60; 95% CI = 0.39–0.92, *P* = 0.019) compared to free/mostly free countries. These results remained significant after adjusting for several sociodemographic and lifestyle covariates.

**Conclusions:**

Both individual and contextual factors seem to have a crucial role in the prevalence of excess weight and obesity in adolescents.

Numerous studies indicate the association and disparities between economic growth and obesity [1–3]. For instance, using data from 67 countries, Pampel et al. [3], explored the linkages between economic development, economic growth, and overweight or obesity (i.e. excess weight) and found that a higher socioeconomic status (SES) was positively associated with excess weight in low-income countries, but the relationship becomes negative in high-income countries. According to Seydel et al. [4], economic expansion in emerging nations is associated with an increase in rates of excess weight. Specifically, a 1% increase in income leads to an increase in the prevalence of excess weight of approximately 0.2 and 0.3%, respectively [4]. Economic and social progress can lead to better health, but it can also increase obesity and widen socioeconomic gaps in obesity rates [3]. In 2016, more than two billion people worldwide had excess weight, and more than 70% of them lived in low- or middle-income countries [5], which supports the idea that financial constraints play a role in increasing the prevalence of obesity among low-income people [6].

Furthermore, the association between family wealth and excess weight is complex and varies according to different studies. For instance, living in a single-parent household, as well as living with grandparents, is related to excess weight in adolescents [7]. Another study reported associations between family wealth and obesity risk behaviours among Canadian adolescents [8]. In a prospective cohort study, poverty status was found to be the strongest predictor of obesity in youth over time [9]. A review article suggests that the association between household income and weight gain is unclear and varies across countries [10]. In addition, non-whites and those with a higher birth weight have been related to greater odds of having obesity [9]. The Family Affluence Scale (FAS) is widely used in research studies to measure adolescent family material wealth [11]. It is a self-reported measure that has been validated in many countries, such as the Czech Republic, Sweden, and the UK [12–14]. It is also used as an indicator of absolute level of socioeconomic position. FAS is recognised as a useful tool for identifying socioeconomic inequalities in adolescent health and well-being [11]. The FAS has been found to be a valid measure of national wealth as it correlates with other measures of national wealth, such as gross domestic product (GDP) [12]. This tool is a simple and cost-effective way to measure household wealth and SES [12]. However, it is crucial to keep in mind that this scale is a self- reported measure and may be subject to bias and measurement error [13].

In the scientific literature, it is shown that economic growth and economic freedom are associated [15,16]. An increase in economic freedom is strongly correlated with economic growth, according to a study using various measures of economic freedom to demonstrate this [17]. In addition, a previous study reported, based on a robust dynamic panel framework, that improvements in economic freedom, as measured by the Heritage Foundation's IEF, are associated with economic growth [15]. Furthermore, panel data analysis of findings from a different study revealed that economic institutions, in particular economic freedom, have a substantial influence on economic development on their own and that total IEF is reliably connected to growth in Middle East and North African (MENA) nations [16]. The fundamentals of economic freedom include voluntary trade enabled by markets, personal choice, the ability to participate and compete in markets, and the protection of people and their property from external violence. The fundamental role of government in an economically free society is to safeguard citizens and their property from external attack [18]. There is also evidence that economic freedom has a greater impact on growth outcomes than culture, suggesting that the two may be interchangeable [19].

Although the scientific literature contains numerous studies on the association between individual economic factors (for example, socioeconomic family status) and excess weight in both the general population and young population [20,21], the association between contextual economic factors has been less studied (especially in adolescents). In this sense, it has been pointed out that neighbourhood SES disparity might be contributing to the burden of excess weight [22]. However, to our knowledge, no previous study has examined the role of IEF in the prevalence of excess weight and obesity in adolescents. This study aims to determine the association between individual and contextual economic factors and the prevalence of excess weight and obesity in adolescents from different countries.

## METHODS

### Study design and population

This cross-sectional study included data from the following countries (Albania, Armenia, Austria, Azerbaijan, Belgium (Flanders), Belgium (Wallonia), Bulgaria, Canada, Switzerland, Czech Republic, Germany, Denmark, Estonia, Spain, France, England, Scotland, Wales, Georgia, Greenland, Greece, Croatia, Hungary, Ireland, Israel, Iceland, Italy, Kazakhstan, Lithuania, Luxembourg, Latvia, Moldova North Macedonia, Malta, the Netherlands, Norway, Poland, Portugal, Romania, Serbia, Russia, Sweden, Slovenia, Slovakia, Turkey, and Ukraine) from the 2017/2018 wave of the HBSC study, covering nationally representative samples of adolescents aged 10–17 years [23]. After excluding countries and participants with missing data, the total number of adolescents included in the analyses was 143 160 (51.7% girls, age range from 10 to 17 years) from the aforementioned countries. Detailed information on the selection of the final sample can be found in Figure S1 in the **Online Supplementary Document****.** The adolescents were randomly selected from their schools and anonymously completed a standardised test, which was conducted in their native language. Students were allowed to leave questions unanswered. Institutional ethics clearance was obtained from each participating country. In addition, schools, adolescents and their parents or legal guardians signed written informed consent forms. Given that the present study was a secondary analysis of anonymised data, the approval of an Ethics Committee was not required.

### Procedures

#### Socioeconomic status (independent variable)

SES was evaluated using the Family Affluence Scale-III (FAS-III) [14], which consists of six questions with responses ranging from 0 to 13 points. The scores were added together to calculate the FAS-III score, where a higher score represents a higher SES. The FAS-III includes six questions on family material assets: number of bathrooms (0, 1, 2, 3 or more), number of cars (0, 1, 2, 3 or more), non-shared bedroom (yes/no), dishwasher (yes/no), number of computers (0, 1, 2, 2 or more), and number of foreign vacations taken in the last twelve months (0, 1, 2, 3 or more). In accordance with international norms, age-group and sex-specific *ridit* scores should be determined for each country participating in the HBSC [12]. *Ridit* scores are then used to define groups of children and adolescents in the bottom 20% (poor wealth), the middle 60% (middle wealth), and the top 20% (high wealth). This suggests that the following thresholds should be applied to both boys and girls in this sample on their SES: low SES (0–7 points), medium SES (8–11 points) and high SES (12–13 points). All items are assigned a score between 0 and 3. Total scores are calculated by summing the total of each score. Low (0–2 points), medium (3–5 points), and high (6 or more points) scores are assigned [24].

#### Index of economic freedom (independent variable)

The index examines 12 quantitative and qualitative economic factors in 184 countries, including rule of law (property rights, government integrity, judicial effectiveness), government size (government spending, tax burden, fiscal health), regulatory efficiency (business freedom, labour freedom, monetary freedom), and open markets (trade freedom, investment freedom, financial freedom) [25]. Its framework comprises four pillars that aggregate a total of 10 categories of economic freedom. The 12 parameters were weighted equally and rated on a scale of zero to one hundred to determine the IEF [26]. The ranking is based on each country and each year of HBSC data collection (i.e. 2017, 2018 or 2019). The index of economic freedom is divided into five categories: repressed (0–49.9 points), mostly unfree (50–59.9 points), moderately free (60-69.9 points), mostly free (70–79.9 points), or free (80–100 points). No country in the HBSC study was found to be in the ‘repressed’ category. Since only two countries out of 46 were in the ‘free’ category, the ‘mostly free’ and ‘free’ categories were combined. For our purposes, England, Wales, and Scotland were assigned the IEF of the UK since there is no individual information on these countries.

#### Excess weight or obesity (dependent variable)

Body weight and height were self-reported by adolescents, which were used to determine body mass index (BMI) (kg/m^2^). Subsequently, BMI z-score values were determined following the International Obesity Task Force (IOTF) criteria [27]; consequently, the prevalence of excess weight (+1.31 standard deviations (SDs) for boys and +1.24 SDs for girls) or obesity (+2.29 SDs for boys and + 2.19 SDs for girls) was calculated.

### Covariates

The adolescents reported their own age and sex. Physical activity was measured with the following question, ‘In a typical or usual week, how many days do you engage in physical activity for at least 60 minutes a day?’ (responses ranged from zero to seven days a week). The frequency of different eating habits was assessed by the questions, ‘How many times a week do you eat fruits/vegetables/sweets/soft drinks?’ (response options: never/less than once a week/two to four times a week/five to six times a week/once a day/more than once a day); ‘How often do you usually eat breakfast (more than one glass of milk or fruit juice)?’ (response options: weekdays: never/one day/two days/three days/four days/five days; weekends: never/only on one day/on both days); and ‘How often do you and your family usually have meals together?’ (response options: every day/most days/about once a week/less often/never). Alcohol and tobacco consumption during the last 30 days were assessed using the next question: ‘On how many days (if any) have you drunk alcohol/smoked cigarettes?)’ (response options: never/1–2 days/3–5 days/6–9 days/10–19 days/20–29 days/30 days or more). For the purpose of this study, these variables were dichotomised as follows: a) fruits, vegetables, sweets, soft drinks, and breakfast consumption as daily or not daily consumption; b) family meals together, as ‘most of the days’ (every day or most days) or ‘not most of the days’ (about once a week, less often, or never); and c) alcohol and tobacco consumption during the last 30 days, as ‘yes’ or ‘no’. Lastly, year of the data collection (i.e. 2017, 2018, or 2019) and mode of survey administration (i.e. pen and pencil or computer) were also considered.

### Statistical analysis

Maps showing the mean (m) FAS-III score and IEF score by country, as well as the prevalence of excess weight and obesity in the different countries were included. Weighted descriptive data were presented as absolute and relative frequencies for categorical variables or m and SD for continuous variables. To examine the association between the mean FAS-III and IEF scores of countries and the prevalence of excess weight or obesity in adolescents, scatter plots were generated. Smoothed lines were incorporated into the scatter plots using the loess method. This locally weighted regression technique was employed to capture nonlinear trends in the data. Following a previously described methodology [28], stepwise models were constructed to better analyse the associations of the independent variables on excess weight or obesity in adolescents. Model 1 included individual sociodemographic variables. Moreover, Model 2 added the specific contextual variable (IEF status) and the random effects (country). The dichotomous measures of risk factors were analysed by logistic regression and the dichotomous form of the dependent variable (excess weight or obesity). In addition, the area under the curve (AUC) in the logistic regression models was calculated to provide information on the ability of the model to correctly classify adolescents with or without co-occurrence of economic risk factors. Differences between specific and general contextual factors were considered. Furthermore, odds ratios (ORs) and beta coefficients of the specific contextual variable were examined. To assess general contextual effects, we estimated the intraclass correlation coefficient (ICC), which represents the proportion of the total individual variance explained by the country of residence, and the median odds ratio (MOR) [29]. The 80% interval odds ratio (IOR-80%) was also determined, which is defined as the middle 80% interval of the OR distribution formed by performing a pairwise random comparison between the IEF classification exposed and unexposed to the contextual variable. The IOR-80% interval is narrow if the between-country variance as a function of IEF is small, and is wide if the between-country variance as a function of IEF is large [29]. If the interval (IOR-80%) contains 1, then for some countries the association is in the opposite direction to the overall ORs. Moreover, the proportion of opposite odds ratios (POOR) was also calculated, which indicates the proportion of ORs with opposite direction to the overall OR [28]. POOR values range from 0 to 50%. A POOR of 0% means that all ORs have the same sign. A POOR of 50% means that half of the ORs are of opposite sign, so the association is very heterogeneous. To measure the goodness of fit of our models, we used the Bayesian information criterion (BIC) [30]. Moreover, Nakagawa’s marginal and conditional coefficients of determination (*R*^2^) were calculated to obtain the variance explained including only fixed effects or both fixed and random effects, respectively [31]. Additionally, predicted probabilities of having excess weight or obesity in adolescents according to FAS score or IEF score (both as continuous variables) were estimated. Statistical significance was set at a *P*-value of 0.05. All analyses accounted for survey weights and were conducted using the software R (Version 4.3.0) (R Core Team, Vienna, Austria) and RStudio (Version 2023.03.1) (Posit, Boston, MA, USA).

## RESULTS

**Figure 1:**
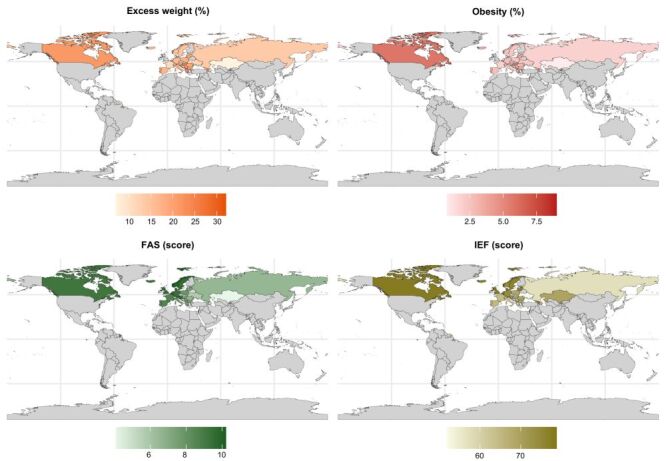
Country means for Family Affluence Scale score, Index of Economic Freedom, prevalence of excess weight and obesity in the 10–17-year-old population of HBSC study, 2017–2018.

**Figure 2:**
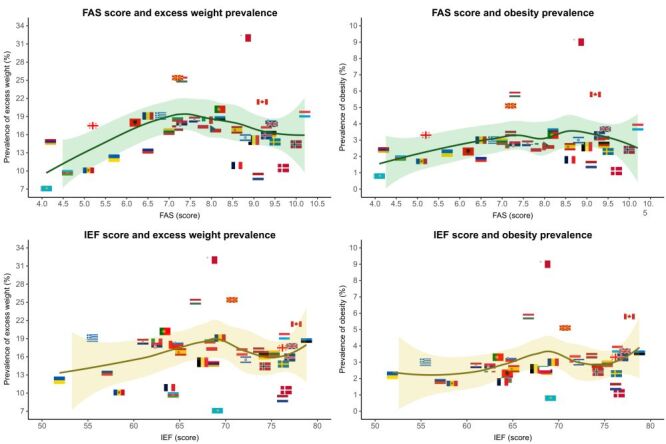
Scatter plots illustrating the associations between Family Affluence Scale or index of economic freedom and excess weight or obesity prevalence in the 10–17-year-old population of HBSC study, 2017–2018.

The prevalence of excess weight and obesity in participants, along with the country-specific mean FAS and IEF scores, is depicted in **Figure 1**. Malta showed the highest excess weight prevalence, reaching 32.0%, while Kazakhstan reported the lowest at 7.1%. Concerning obesity prevalence, Malta exhibited the highest rate at 9.0% and Kazakhstan the lowest rate at 0.8%. On the other hand, Luxembourg had the highest mean FAS score (m = 10.2; SD = 2.1), while Kazakhstan reports the lowest mean FAS score (m = 4.1; SD = 2.4). With regards to IEF, the Estonia reported the highest index (78.8), while Slovenia recorded the lowest index (51.9). **Figure 2** displays scatter plots illustrating the associations between FAS scores and excess weight prevalence (top left), FAS scores and obesity prevalence (top right), IEF scores and excess weight prevalence (bottom left), and IEF scores and obesity prevalence (bottom right). Each point (i.e. flag) on the plots represents a country, with loess smoothing lines providing a visual trend of the nonlinear associations. Smoothed trend curves show some upward (though not linear) trajectory in the relationship between countries' FAS-III or IEF scores and the prevalence of adolescent excess weight or obesity in those countries.

**Table 1** and **Table 2** show the descriptive data of the sample of adolescents examined according to their SES or IEF status, respectively. Overall, the prevalence of excess weight and obesity were 15.8 and 2.9%, respectively. Regarding SES, adolescents with a low SES showed the highest prevalence of excess weight and obesity (excess weight = 20.5%; obesity = 4.2%), while the lowest prevalence were found in those with a high SES (excess weight = 13.2%; obesity = 2.0%). Concerning IEF, the lowest prevalence of adolescents with excess weight and obesity were observed in mostly unfree countries (excess weight = 13.4%; obesity = 2.0%). Conversely, the highest prevalence of adolescents with excess weight and obesity were found in free/mostly free countries (excess weight = 17.3%; obesity = 3.4%).

**Table 1:** Weighted characteristics of study participants according to socioeconomic status

Variables*	Low SES	Medium SES	High SES
Sex			
*Male*	12 488 (47.6)	43 404 (48.5)	13 727 (49.2)
*Female*	13 736 (52.4)	46 039 (51.5)	14 197 (50.8)
Age, mean (SD)	13.6 (1.6)	13.6 (1.6)	13.6 (1.7)
Physical activity (days), mean (SD)†	3.8 (2.1)	4.1 (2.0)	4.5 (2.0)
Fruits consumption			
*Not daily*	16 975 (64.7)	53 547 (59.9)	14 191 (50.8)
*Daily*	9249 (35.3)	35 896 (40.1)	13 733 (49.2)
Vegetables consumption			
*Not daily*	17 139 (65.4)	54 935 (61.4)	14 920 (53.4)
*Daily*	9085 (34.6)	34 508 (38.6)	13 004 (46.6)
Breakfast consumption			
*Not daily*	14 169 (54.0)	42 042 (47.0)	11 937 (42.7)
*Daily*	12 055 (46.0)	47 401 (53.0)	15 987 (57.3)
Sweets consumption			
*Not daily*	19 828 (75.6)	67 910 (75.9)	20 885 (74.8)
*Daily*	6396 (24.4)	21 533 (24.1)	7039 (25.2)
Soft drinks consumption			
*Not daily*	21 827 (83.2)	76 717 (85.8)	24 081 (86.2)
*Daily*	4397 (16.8)	12 726 (14.2)	3843 (13.8)
Family meals			
*No most of days*	5699 (21.7)	15 097 (16.9)	4064 (14.6)
*Most of days*	20 525 (78.3)	74 346 (83.1)	23 860 (85.4)
Alcohol consumption†			
*No*	21 393 (81.6)	71 109 (79.5)	21 524 (77.1)
*Yes*	4831 (18.4)	18 334 (20.5)	6400 (22.9)
Tobacco consumption‡			
*No*	24 178 (92.2)	83 359 (93.2)	25 892 (92.7)
*Yes*	2046 (7.8)	6084 (6.8)	2032 (7.3)
Year of data collection			
*2017*	3840 (14.6)	12 620 (14.1)	3954 (14.2)
*2018*	22 285 (85.0)	76 512 (85.5)	23 904 (85.6)
*2019*	99 (0.4)	342 (0.4)	80 (0.3)
Mode of survey administration			
*Pen and paper*	13 964 (53.3)	48 274 (54.0)	15 217 (54.5)
*Computer*	12 259 (46.7)	41 201 (46.0)	12 721 (45.5)
Excess weight§			
*No*	21 847 (79.5)	74 759 (83.6)	24 245 (86.8)
*Yes*	5377 (20.5)	14 716 (16.4)	3694 (13.2)
Obesity§			
*No*	25 124 (95.8)	86 840 (97.1)	27 368 (98.0)
*Yes*	1100 (4.2)	2634 (2.9)	570 (2.0)

SD – standard deviation, SES – socioeconomic status

*All values are expressed as %, unless otherwise indicated.

†At least 60 min/d.

‡During the last 30 d.

§According to the International Obesity Task Force criteria [27].

**Table 2:** Weighted characteristics of study participants according to economic freedom status

Variables*	Mostly free/free	Moderately free	Mostly unfree
Sex			
*Male*	32 510 (49.1)	29 956 (48.0)	7195 (47.9)
*Female*	33 676 (50.9)	32 460 (52.0)	7840 (52.1)
Age, mean (SD)	13.7 (1.6)	13.6 (1.6)	13.7 (1.7)
Physical activity (days), mean (SD)†	4.2 (2.0)	4.1 (2.1)	3.9 (2.1)
Fruits consumption			
*Not daily*	39 083 (59.1)	36 660 (58.7)	8998 (59.8)
*Daily*	27 103 (40.9)	25 756 (41.3)	6037 (40.2)
Vegetables consumption			
*Not daily*	39 832 (60.2)	38 485 (61.7)	8704 (57.9)
*Daily*	26 354 (39.8)	23 931 (38.3)	6331 (42.1)
Breakfast consumption			
*Not daily*	31 208 (47.2)	29 853 (47.8)	7106 (47.3)
*Daily*	34 978 (52.8)	32 562 (52.2)	7929 (52.7)
Sweets consumption			
*Not daily*	52 447 (79.2)	45 487 (72.9)	10 724 (71.3)
*Daily*	13 739 (20.8)	16 928 (27.1)	4312 (28.7)
Soft drinks consumption			
*Not daily*	58 066 (87.7)	51 052 (81.8)	13 546 (90.1)
*Daily*	8119 (12.3)	11 364 (18.2)	1489 (9.9)
Family meals			
*No most of days*	12 866 (19.4)	9914 (15.9)	2085 (13.9)
*Most of days*	53 319 (80.6)	52 502 (84.1)	12 951 (86.1)
Alcohol consumption‡			
*No*	52 587 (79.5)	49 195 (78.8)	12 278 (81.7)
*Yes*	13 598 (20.5)	13 221 (21.2)	2758 (18.3)
Tobacco consumption‡			
*No*	61 642 (93.1)	57 723 (92.5)	14 102 (93.8)
*Yes*	4543 (6.9)	4692 (7.5)	934 (6.2)
Year of data collection			
*2017*	10 160 (15.4)	10 254 (16.4)	–
*2018*	55 871 (84.4)	51 795 (83.0)	15 035 (100.0)
*2019*	155 (0.2)	367 (0.6)	–
Mode of survey administration			
*Pen and paper*	31 959 (48.3)	31 766 (50.9)	13 730 (91.3)
*Computer*	34 226 (51.7)	30 650 (49.1)	1305 (8.7)
Excess weight§			
*No*	54 730 (82.7)	52 098 (83.5)	13 022 (86.6)
*Yes*	11 455 (17.3)	10 317 (16.5)	2014 (13.4)
Obesity§			
*No*	63 935 (96.6)	60 688 (97.2)	14 710 (97.8)
*Yes*	2251 (3.4)	1728 (2.8)	325 (2.2)

SD – standard deviation

*All values are presented as %, unless otherwise indicated.

†At least 60 min/d.

‡During the last 30 d.

§According to the International Obesity Task Force criteria [27].

The results of different logistic regression models that were used to analyse the relationship between individual and contextual economic factors and excess weight or obesity in adolescents, after adjusting for several covariates are shown in **Table 3** and **Table 4**, respectively. An inverse association was shown between SES and excess weight or obesity, with adolescents with high SES and medium SES being less likely to have excess weight compared to adolescents with low SES (medium SES: OR = 0.79; 95% confidence interval (CI) = 0.77–0.82, *P* < 0.001), (high SES: OR = 0.65; 95% CI = 0.62–0.68, *P* < 0.001). For obesity, lower odds were also found for adolescents with medium SES (medium SES: OR = 0.74; 95% CI = 0.69–0.80, *P* < 0.001) or high SES (high SES: OR = 0.55; 95% CI = 0.49–0.61, *P* < 0.001), in comparison with their counterparts with low SES. On the other hand, it was observed a lower likelihood of having excess weight (barely significant) and obesity in mostly unfree countries (excess weight: OR = 0.72; 95% CI = 0.51–1.00, *P* = 0.052), (obesity: OR = 0.60; 95% CI = 0.39–0.92, *P* = 0.019) compared to free/mostly free countries. Moreover, the MOR indicated a higher probability of having excess weight (MOR = 1.34; 95% CI = 1.03–1.52) or obesity (MOR = 1.42; 95% CI = 1.16–1.61) if an adolescent moved from a mostly unfree country to a free/mostly free country. Lastly, the POOR indicated that, when comparing countries with different IEF status (i.e. free/mostly free vs. mostly unfree), approximately 25% (for excess weight) and 18% (for obesity) had ORs in the opposite direction.

**Table 3:** Multilevel logistic regression analysis of having excess weight in the 10–17-y-old population of HBSC study, 2017–2018*

	Excess weight	
	**Simple logistic regression analysis**	**Multilevel logistic regression analysis**
	**Model 1‡**	**Model 2§**
Specific individual average effects		
*Low SES*	Reference	Reference
*Medium SES*	0.79 (0.76–0.82, *P* < 0.001)	0.79 (0.77–0.82, *P* < 0.001)
*High SES*	0.63 (0.61–0.67, *P* < 0.001)	0.65 (0.62–0.68, *P* < 0.001)
Specific contextual average effects		
*Free/mostly free countries*		Reference
*Moderately free countries*		1.01 (0.82–1.23, *P* = 0.948)
*Mostly unfree countries*		0.72 (0.51–1.00, *P* = 0.052)
*80% IOR† (free/mostly free vs. moderately free)*		0.58–1.78
*80% IOR† (free/mostly free vs. mostly unfree)*		0.42–1.30
*POOR, % (free/mostly free vs. moderately free)*		48
*POOR, % (free/mostly free vs. mostly unfree)*		25
General contextual effects		
*Country variance*		0.096 (0.001–0.191)
*ICC, %*		2.84 (1.03–5.48)
*MOR*		1.34 (1.03–1.52)
*AUC*	0.645 (0.641–0.649)	0.645 (0.641–0.649)
*AUC change*		<0.001
Model performance metrics		
*BIC*	122844.5	122865.0
*BIC change*		−20.5
*Nakagawa’s R^2^*	0.080	0.081

AUC – area under the curve, BIC – Bayesian information criterion, ICC – interclass correlation coefficient, MOR – median odds ratio, POOR – proportion of opposite odds ratios, R^2^ – coefficients of determination, SES – socioeconomic status

*Values are expressed as odds ratios (OR) with 95% confidence interval (CI), unless otherwise indicated. The constant is not shown in the Table.

†80% IOR stands for 80% mean interval of the OR distribution.

‡Model 1: adjusted for age, sex, physical activity, fruit, vegetables, soft drinks, sweets, breakfast consumption, family meals, year of data collection, and mode of survey administration.

§Model 2: Model 1 + specific contextual average effects (economic freedom index) + general contextual effects (country).

**Table 4:** Multilevel logistic regression analysis of having obesity in the 10–17-y-old population of HBSC study, 2017–2018

	Obesity	
	**Simple logistic regression analysis**	**Multilevel logistic regression analysis**
	**Model 1‡**	**Model 2§**
Specific individual average effects		
*Low SES*	Reference	Reference
*Medium SES*	0.68 (0.63–0.73, *P* < 0.001)	0.75 (0.69–0.80, *P* < 0.001)
*High SES*	0.46 (0.42–0.51, *P* < 0.001)	0.55 (0.50–0.61, *P* < 0.001)
Specific contextual average effects		
*Free/mostly free countries*		Reference
*Moderately free countries*		0.88 (0.69–1.14, *P* = 0.336)
*Mostly unfree countries*		0.60 (0.39–0.92, *P* = 0.019)
*80% IOR† (free/mostly free vs. moderately free)*		0.46–1.75
*80% IOR† (free/mostly free vs. mostly unfree)*		0.32–1.22
*POOR (%) (free/mostly free vs. moderately free)*		42
*POOR (%) (free/mostly free vs. mostly unfree)*		18
General contextual effects		
*Country variance*		0.137 (0.024–0.250)
*ICC (%)*		4.00 (0.72–7.06)
*MOR*		1.42 (1.16–1.61)
*AUC*	0.686 (0.679–0.694)	0.688 (0.680–0.695)
*AUC change*		0.001
Model performance metrics		
*BIC*	36671.6	36690.8
*BIC change*		−19.27
*Nakagawa’s R^2^*	0.122	0.121

AUC – area under the curve, BIC – Bayesian information criterion, ICC – interclass correlation coefficient, MOR – median odds ratio, POOR – proportion of opposite odds ratios, R^2^ – coefficients of determination, SES – socioeconomic status

*Values are expressed as odds ratios (OR) with 95% confidence interval (CI), unless otherwise indicated. The constant is not shown in the Table.

†80% IOR, 80% mean interval of the OR distribution.

‡Model 1: adjusted for age, sex, physical activity, fruit, vegetables, soft drinks, sweets, breakfast consumption, family meals, year of data collection, and mode of survey administration.

§Model 2: Model 1 + specific contextual average effects (economic freedom index) + general contextual effects (country).

**Figure 3:**
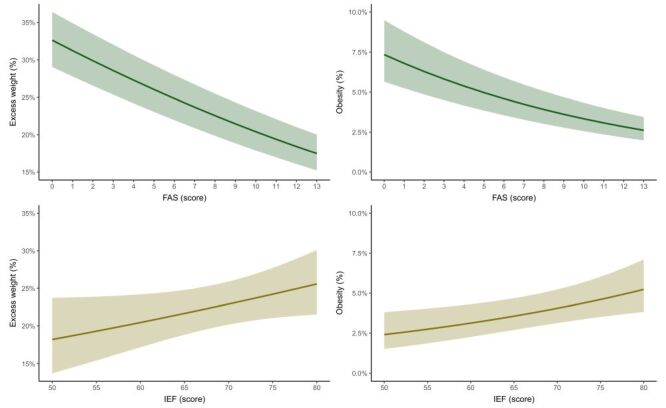
Predicted probabilities of having excess weight or obesity among adolescents according to Family Affluence Scale score or index of economic freedom score in the 10–17-year-old population of HBSC study, 2017–2018.

**Figure 3** displays the predicted probabilities of having excess weight or obesity in adolescents according to FAS score or IEF score (both as continuous variables). For each additional point on the FAS, the probability of having overweight and obesity adolescents were lower (excess weight = −0.84%; 95% CI = −0.94 to −0.74%, *P* < 0.001), (obesity = −0.23%; 95% CI = −0.28 to −0.19%, *P* < 0.001). Conversely, for each additional point on the IEF, the probability of having obesity in adolescents was higher (0.08%; 95% CI = 0.02–0.13%, *P* = 0.008). For excess weight, the probability was also greater but a with a near-significant trend (0.19%; 95% CI = −0.01–0.39%, *P* = 0.059).

## DISCUSSION

To our knowledge, this is the first study considering the role of IEF in the association between economic factors and excess weight and obesity in adolescents. Overall, even adjusting for several sociodemographic (i.e. age and sex) and lifestyle variables (i.e. eating habits, physical activity, drug consumption), our findings showed that the prevalence of excess weight and obesity in adolescents was related to both individual and contextual economic factors. More specifically, it was observed that the probability of having excess weight or obesity was lower when SES was higher. Conversely, in relation to the economic context (measured through the IEF), our findings showed an association between higher IEF and greater probabilities of having excess weight or obesity. Notwithstanding, caution is required to interpret this result due to the heterogeneity observed among countries. Although the associations of IEF with excess weight or obesity in adolescents have been less studied in the literature, there are some possible pathways that could be helpful for understanding these findings.

In relation to SES, we observed a lower probability of having excess weight and obesity in adolescents with a medium SES and a high SES compared to those with a low SES. This result is consistent with the scientific literature [20,21]. The association between household SES and the prevalence of excess weight and obesity in adolescents may be due to a variety of interrelated factors such as access to healthy foods, as higher SES households tend to have greater economic resources to purchase nutritious and quality foods [32]. These households can access a greater variety of fruits, fresh vegetables and lean proteins, which contributes to a lower energy intake and, therefore, a lower probability of having excess weight [33]. In contrast, lower SES families may face economic constraints that restrict their access to healthy food options [34]. In addition, the environment in which adolescents live may influence their food choices and physical activity levels [35]. Areas with a higher SES may promote better health by attracting certain institutions (e.g. grocery stores, places to exercise), offering physical features that are conducive to physical activity (e.g. food stores, places to exercise), offering physical features that are conducive to physical activity (e.g. well-maintained buildings, parks, and streets) and encourage a set of norms that emphasise healthy behaviours (e.g. exercise and good nutrition) [36]. In contrast, low SES neighbourhoods have been associated with greater availability of fast and energy-dense food outlets, along with lower availability of fruit and vegetable outlets and limited sports facilities [22]. Socioeconomic inequalities are also associated with stress and mental health problems. Lower SES families may face higher levels of financial stress, job insecurity, and general socioeconomic difficulties. Chronic stress and mental health problems may contribute to increased consumption of unhealthy foods as a coping mechanism, as well as a lack of motivation to maintain an active and healthy lifestyle [37]. Finally, higher SES is often associated with higher levels of education and knowledge about nutrition and the importance of maintaining a healthy lifestyle [38]. Families with a higher SES may have more access to information, educational programmes, and health services that promote healthy habits and prevent excess weight [39]. This greater awareness and knowledge could influence dietary choices and the adoption of healthy behaviours in adolescents.

On the other hand, our results indicated lower odds of having excess weight or obesity in mostly unfree countries in comparison with free/mostly free countries, which is in line with the scientific literature [40]. Although the scientific literature examining the relationship between IEF and excess weight and obesity is scarce (especially in adolescents), one of the main reasons may be the freedom of trade agreements that have an impact on further deregulation of the food sector. Supporting this notion, Barlow et al. [41] revealed that free trade agreements pose significant health risks, since these trade agreements are associated with both increased consumption of sugar-sweetened beverages and processed foods. Furthermore, the policies associated with these trade agreements have been shown to be associated with higher BMI [42]. This liberalisation of the sector could also be related to a greater offer of access to high-density foods through different promotion and advertising channels, as well as to greater economic insecurity, whereas, in countries with a liberal market approach, there is often a significant gap between high and low incomes [43]. This can lead to an unequal distribution of wealth and increase economic insecurity for those at the bottom of the income ladder, in addition these countries are exposed to increased international competition, which can lead to job losses and economic insecurity when companies face pressure to reduce costs and maintain their competitiveness [44]. Supporting this notion, Offer et al. [45], indicated that regulations and trade can affect the quantity and quality of food available to consumers through new food technologies, marketing and competition. Similarly, Smith et al. [46] reported that the perception of economic insecurity, such as the risk of unemployment or other loss of income, generates stress, could lead to overeating. Finally, Ljungvall [47] pointed out that an environment with more economic freedom can encourage unhealthy behaviours by affecting the quality and quantity of food available to consumers, affecting access to safety nets and access to environments for physical activity, leading to increases in BMI. All the factors previously raised could, at least partially, explain the association between a higher IEF and a higher prevalence of excess weight and obesity in adolescents.

Despite the findings obtained for our main independent variables examined, we should consider that the sociodemographic and lifestyle variables adjusted in our analyses also showed association with excess weight and obesity in adolescents. For instance, the results revealed that girls are less likely to have excess weight or obesity than boys. Previous studies have shown that sex may be related to body weight and body composition patterns, with girls showing a greater concern for body image and a greater tendency to engage in weight loss behaviours [48,49]. This tendency may be biologically caused because women tend to have a higher proportion of body fat and a lower basal metabolism compared to men, which may influence the prevalence of excess weight and obesity. In addition, sociocultural factors such as social pressure and beauty ideals may lead women to be more conscious of their body image and adopt behaviours related to weight loss. On the other hand, we observed that a higher number of days spent in physical activity was related to lower odds of having excess weight and obesity. This finding is in line with the scientific literature that indicates the benefits of engaging in sufficient physical activity to reduce measures of adiposity [50]. Similarly, the likelihood of having excess weight tends to increase with age due to changes in physical activity (among other factors) [51]. Furthermore, while some research has found that young people with excess weight tend to be less physically active than youth of a healthier weight [52,53], this may be due to several factors, such as lack of confidence in their physical abilities, fear of weight-related stigma or discrimination, or lack of motivation. Additionally, certain eating habits such as daily vegetable consumption also decrease the likelihood of having excess weight, which is consistent with the scientific literature [54–56]. Studies indicate that the habit of eating breakfast together with daily moderate-vigorous physical activity seems to reduce the likelihood of having excess weight [57,58], this could be a consequence of the fact that vegetables provide nutrients and fibre that promote satiety and help control caloric consumption. Similarly, daily breakfast provides energy and prevents feelings of hunger during the day, improving appetite and satiety and can be an important strategy to improve diet quality [59].

The results of this study should be interpreted considering certain limitations. First, due to the cross-sectional design of the study, we cannot establish a cause-effect relationship for the observations found. Therefore, future prospective observational studies based on objective measures are required to examine whether higher SES or higher IEF leads to a lower or higher risk of presenting excess weight or obesity in adolescents. Second, although several of the measures used have been previously validated, the questions asked were brief (intended to reduce the question burden on participants) and did not provide in-depth data on the variables examined. A more detailed measure would provide additional information for each item, as well as information on other aspects related to economics. Third, body weight and height were reported by the adolescents, which could introduce errors and biases in the results obtained. In addition, information on lifestyle and excess weight or obesity may lead to some differential bias due to information and recall bias, social desirability bias, or overestimation by adolescents. Fourth, although we controlled in our analysis for the effect of important confounding factors, including sociodemographic (sex and age) and lifestyle variables (eating habits, physical activity, drug consumption), it is still possible that there are residual confounding factors. In contrast, this study presents several strengths at the methodological level. The main strength of the study is the large and representative sample of adolescents analysed from 41 countries, which confers substantial external validity to the findings obtained. Although the statistical significance of small effect sizes may appear in studies analysing large data sets, the results of this study provide evidence of the association of individual and contextual economic factors with excess weight and obesity in adolescents.

## CONCLUSIONS

Our study offers evidence that both individual and contextual economic factors are associated with the prevalence of excess weight and obesity in adolescents. These findings suggest an inverse association between SES and excess weight and obesity, where adolescents of lower SES seem to have a higher probability of having excess weight and obesity compared to those of higher SES. Furthermore, adolescents from mostly unfree countries had a lower likelihood of having excess weight and obesity, compared to free/mostly free countries. These results highlight the need to address both individual and contextual factors in the prevention and management of excess weight and obesity in adolescents. Policies need to be implemented that address inequalities in the food environment and opportunities for physical activity in communities with lower socioeconomic levels, which could include promoting the availability and accessibility of healthy foods at affordable prices, as well as the creation of safe and accessible spaces for physical activity.

## Additional material


Online Supplementary Document
