# Supplementary material for: The role of individual and contextual economic factors in obesity among adolescents: A cross-sectional study including 143 160 participants from 41 countries
Source: J Glob Health. 2024 Feb 23;14:04035. doi: 10.7189/jogh.14.04035 (PMC10884718; doi:10.7189/jogh.14.04035)
Supplement: Online Supplementary Document [file jogh-14-04035-s001.pdf]

Figure S1. Flow chart for the selection of the participants.

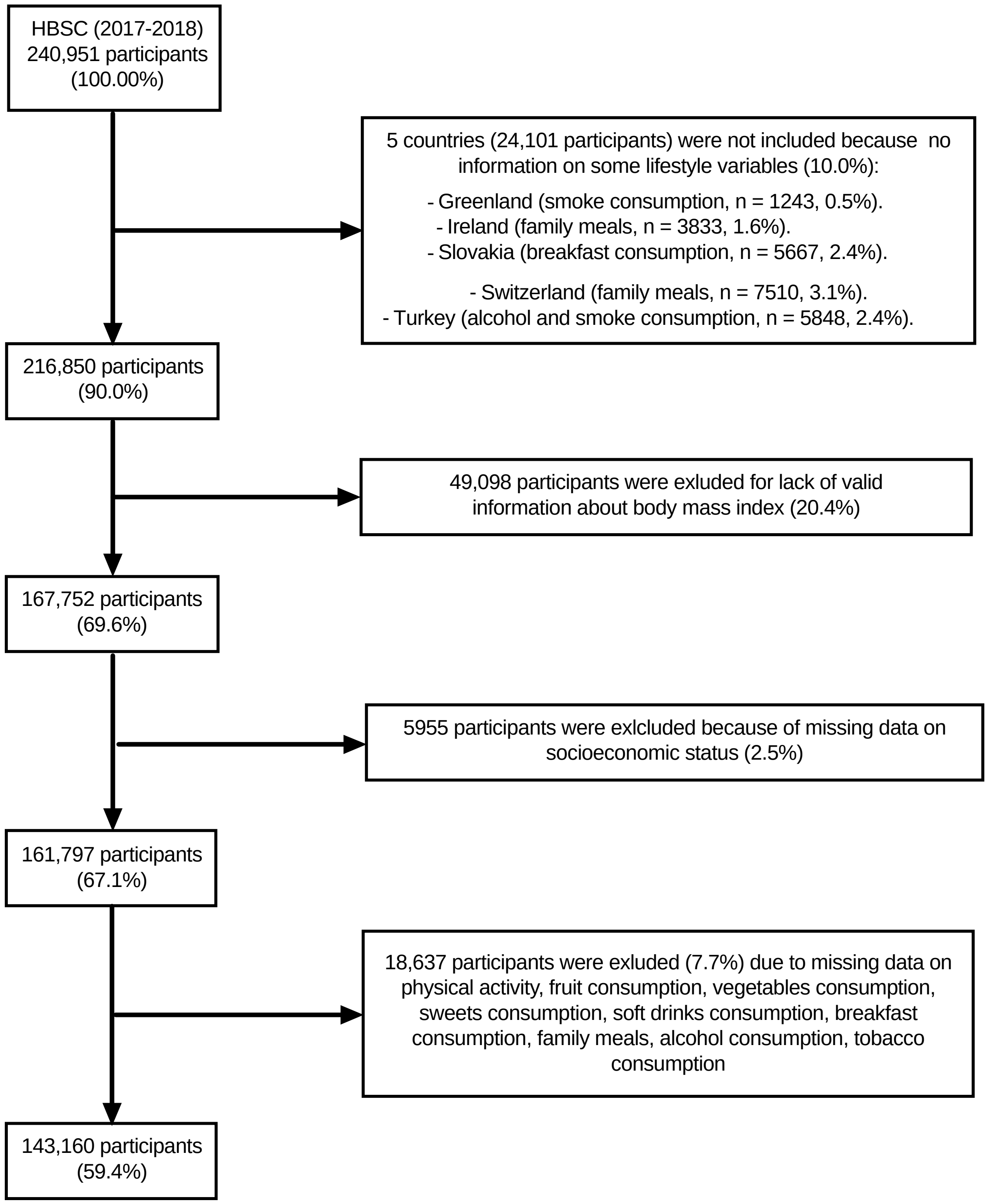

Table S1. Full multilevel logistic regression analysis of having excess weight according to socioeconomic status among adolescents.

**Predictors**		**No excess weight** §	**Excess weight** §	**OR (univariable)**	**OR (multilevel)**
Sex	Male	55,602 (80.4)	13,568 (19.6)	Reference	Reference
	Female	63,779 (86.2)	10,211 (13.8)	0.66 (0.64-0.67, p<0.001)	0.61 (0.59-0.62, p<0.001)
Age (years)	Mean (SD)	13.6 (1.6)	13.6 (1.6)	1.01 (1.00-1.01, p=0.197)	0.96 (0.95-0.97, p<0.001)
Physical activity (days) †	Mean (SD)	4.2 (2.1)	3.8 (2.0)	0.92 (0.91-0.93, p<0.001)	0.90 (0.90-0.91, p<0.001)
Fruit consumption	No daily	69,937 (82.6)	14,782 (17.4)	Reference	Reference
	Daily	49,444 (84.6)	8997 (15.4)	0.86 (0.84-0.89, p<0.001)	1.00 (0.97-1.04, p=0.850)
Vegetables consumption	No daily	71,805 (82.5)	15,187 (17.5)	Reference	Reference
	Daily	47,576 (84.7)	8592 (15.3)	0.85 (0.83-0.88, p<0.001)	1.05 (1.01-1.08, p=0.009)
Breakfast consumption	No daily	54,722 (80.5)	13,216 (19.5)	Reference	Reference
	Daily	64,659 (86.0)	10,563 (14.0)	0.68 (0.66-0.70, p<0.001)	0.73 (0.70-0.75, p<0.001)
Sweets consumption	No daily	88,930 (82.3)	19,189 (17.7)	Reference	Reference
	Daily	30,451 (86.9)	4590 (13.1)	0.70 (0.67-0.72, p<0.001)	0.69 (0.66-0.72, p<0.001)
Soft drinks consumption	No daily	101,889 (83.4)	20,258 (16.6)	Reference	Reference
	Daily	17,492 (83.2)	3521 (16.8)	1.01 (0.97-1.05, p=0.538)	1.04 (0.99-1.08, p=0.114)
Family meals together	No most of days	19,725 (79.8)	4978 (20.2)	Reference	Reference
	Most of days	99,656 (84.1)	18,801 (15.9)	0.75 (0.72-0.77, p<0.001)	0.85 (0.82-0.88, p<0.001)
Alcohol consumption ‡	No daily	95,505 (83.8)	18,438 (16.2)	Reference	Reference
	Daily	23,876 (81.7)	5341 (18.3)	1.16 (1.12-1.20, p<0.001)	1.08 (1.03-1.12, p<0.001)
Smoke consumption ‡	No daily	111,234 (83.6)	21,789 (16.4)	Reference	Reference
	Daily	8147 (80.4)	1990 (19.6)	1.25 (1.18-1.31, p<0.001)	1.09 (1.03-1.16, p=0.002)
Year of data collection	2017	17,599 (86.9)	2661 (13.1)	Reference	Reference
	2018	101,396 (82.8)	21,022 (17.2)	1.37 (1.31-1.43, p<0.001)	1.20 (1.05-1.37, p=0.009)
	2019	386 (80.1)	96 (19.9)	1.64 (1.30-2.05, p<0.001)	1.34 (1.02-1.77, p=0.036)
Mode of survey administration	Pen and pencil	64,610 (83.1)	13,096 (16.9)	Reference	Reference
	Computer	54,771 (83.7)	10,683 (16.3)	0.96 (0.94-0.99, p=0.007)	0.88 (0.82-0.95, p=0.001)
FAS status	Low SES	20,914 (79.3)	5468 (20.7)	Reference	Reference
	Medium SES	74,436 (83.6)	14,645 (16.4)	0.75 (0.73-0.78, p<0.001)	0.79 (0.77-0.82, p<0.001)
	High SES	24,031 (86.8)	3666 (13.2)	0.58 (0.56-0.61, p<0.001)	0.65 (0.62-0.68, p<0.001)

Predictors		No excess weight §	Excess weight §	OR (univariable)	OR (multilevel)
IEF status	Mostly Free/Free	53,818 (82.6)	11,373 (17.4)	Reference	Reference
	Moderately Free	52,101 (83.4)	10,334 (16.6)	0.94 (0.91-0.97, p<0.001)	1.01 (0.82-1.23, p=0.948)
	Mostly Unfree	13,462 (86.7)	2072 (13.3)	0.73 (0.69-0.77, p<0.001)	0.72 (0.51-1.00, p=0.052)

FAS, Family Affluence Scale; IEF, index of economic freedom; OR, odds ratio. † At least 60 minutes per day. ‡ During the last 30 days. § Excess weight (overweight or obesity) status according to the International Obesity Task Force criteria [27].

Table S2. Full multilevel logistic regression analysis of having obesity according to socioeconomic status among adolescents.

**Predictors**		**No obesity** [§]	**Obesity** [§]	**OR (univariable)**	**OR (multilevel)**
Sex	Male	66,595 (96.3)	2575 (3.7)	Reference	Reference
	Female	72,248 (97.6)	1742 (2.4)	0.62 (0.59-0.66, p<0.001)	0.56 (0.53-0.60, p<0.001)
Age (years)	Mean (SD)	13.6 (1.6)	13.6 (1.7)	1.01 (0.99-1.03, p=0.426)	0.95 (0.93-0.97, p<0.001)
Physical activity (days) [†]	Mean (SD)	4.1 (2.1)	3.6 (2.1)	0.89 (0.87-0.90, p<0.001)	0.87 (0.86-0.88, p<0.001)
Fruit consumption	No daily	81,959 (96.7)	2760 (3.3)	Reference	Reference
	Daily	56,884 (97.3)	1557 (2.7)	0.81 (0.76-0.87, p<0.001)	1.00 (0.93-1.08, p=0.991)
Vegetables consumption	No daily	84,179 (96.8)	2813 (3.2)	Reference	Reference
	Daily	54,664 (97.3)	1504 (2.7)	0.82 (0.77-0.88, p<0.001)	1.05 (0.97-1.13, p=0.242)
Breakfast consumption	No daily	65,343 (96.2)	2595 (3.8)	Reference	Reference
	Daily	73,500 (97.7)	1722 (2.3)	0.59 (0.55-0.63, p<0.001)	0.66 (0.62-0.70, p<0.001)
Sweets consumption	No daily	104,582 (96.7)	3537 (3.3)	Reference	Reference
	Daily	34,261 (97.8)	780 (2.2)	0.67 (0.62-0.73, p<0.001)	0.67 (0.61-0.73, p<0.001)
Soft drinks consumption	No daily	118,506 (97.0)	3641 (3.0)	Reference	Reference
	Daily	20,337 (96.8)	676 (3.2)	1.08 (0.99-1.18, p=0.064)	1.11 (1.01-1.22, p=0.024)
Family meals together	No most of days	23,705 (96.0)	998 (4.0)	Reference	Reference
	Most of days	115,138 (97.2)	3319 (2.8)	0.68 (0.64-0.74, p<0.001)	0.80 (0.74-0.86, p<0.001)
Alcohol consumption [‡]	No daily	110,602 (97.1)	3341 (2.9)	Reference	Reference
	Daily	28,241 (96.7)	976 (3.3)	1.14 (1.06-1.23, p<0.001)	1.03 (0.95-1.12, p=0.446)
Smoke consumption [‡]	No daily	129,108 (97.1)	3915 (2.9)	Reference	Reference
	Daily	9735 (96.0)	402 (4.0)	1.36 (1.22-1.51, p<0.001)	1.23 (1.09-1.38, p=0.001)
Year of data collection	2017	19,793 (97.7)	467 (2.3)	Reference	Reference
	2018	118,591 (96.9)	3827 (3.1)	1.37 (1.24-1.51, p<0.001)	1.36 (1.06-1.75, p=0.015)
	2019	459 (95.2)	23 (4.8)	2.12 (1.35-3.19, p=0.001)	2.30 (1.37-3.87, p=0.002)
Mode of survey administration	Pen and pencil	75,262 (96.9)	2444 (3.1)	Reference	Reference
	Computer	63,581 (97.1)	1873 (2.9)	0.91 (0.85-0.96, p=0.002)	0.81 (0.70-0.93, p=0.002)
FAS status	Low SES	25,250 (95.7)	1132 (4.3)	Reference	Reference
	Medium SES	86,457 (97.1)	2624 (2.9)	0.68 (0.63-0.73, p<0.001)	0.75 (0.69-0.80, p<0.001)
	High SES	27,136 (98.0)	561 (2.0)	0.46 (0.42-0.51, p<0.001)	0.55 (0.50-0.61, p<0.001)

Predictors		No obesity §	Obesity §	OR (univariable)	OR (multilevel)
IEF status	Mostly Free/Free	62,935 (96.5)	2256 (3.5)	Reference	Reference
	Moderately Free	60,709 (97.2)	1726 (2.8)	0.79 (0.74-0.85, p<0.001)	0.88 (0.69-1.14, p=0.336)
	Mostly Unfree	15,199 (97.8)	335 (2.2)	0.61 (0.55-0.69, p<0.001)	0.60 (0.39-0.92, p=0.019)

FAS, Family Affluence Scale; IEF, index of economic freedom; OR, odds ratio. † At least 60 minutes per day. ‡ During the last 30 days. § Obesity status according to the International Obesity Task Force criteria [27].
